# Role of traditional Chinese medicine in age-related macular degeneration: exploring the gut microbiota’s influence

**DOI:** 10.3389/fphar.2024.1356324

**Published:** 2024-01-25

**Authors:** Yujia Yu, Yong Liu, Zhaoru Meng

**Affiliations:** ^1^ Shandong University of Traditional Chinese Medicine, Jinan, Shandong, China; ^2^ Affiliated Hospital of Shandong University of Traditional Chinese Medicine, Shandong Province Hospital of Traditional Chinese Medicine, Jinan, Shandong, China; ^3^ The Hong Kong University of Science and Technology, Kowloon, Hong Kong SAR, China

**Keywords:** age-related macular degeneration, gut microbiota composition, gut microbiota metabolites, gut-retinal axis, traditional Chinese medicine

## Abstract

The pathogenesis of age-related macular degeneration (AMD), a degenerative retinopathy, remains unclear. Administration of anti-vascular endothelial growth factor agents, antioxidants, fundus lasers, photodynamic therapy, and transpupillary warming has proven effective in alleviating symptoms; however, these interventions cannot prevent or reverse AMD. Increasing evidence suggests that AMD risk is linked to changes in the composition, abundance, and diversity of the gut microbiota (GM). Activation of multiple signaling pathways by GM metabolites, including lipopolysaccharides, oxysterols, short-chain fatty acids (SCFAs), and bile acids (BAs), influences retinal physiology. Traditional Chinese medicine (TCM), known for its multi-component and multi-target advantages, can help treat AMD by altering GM composition and regulating the levels of certain substances, such as lipopolysaccharides, reducing oxysterols, and increasing SCFA and BA contents. This review explores the correlation between GM and AMD and interventions for the two to provide new perspectives on treating AMD with TCM.

## 1 Introduction

**FIGURE 1:**
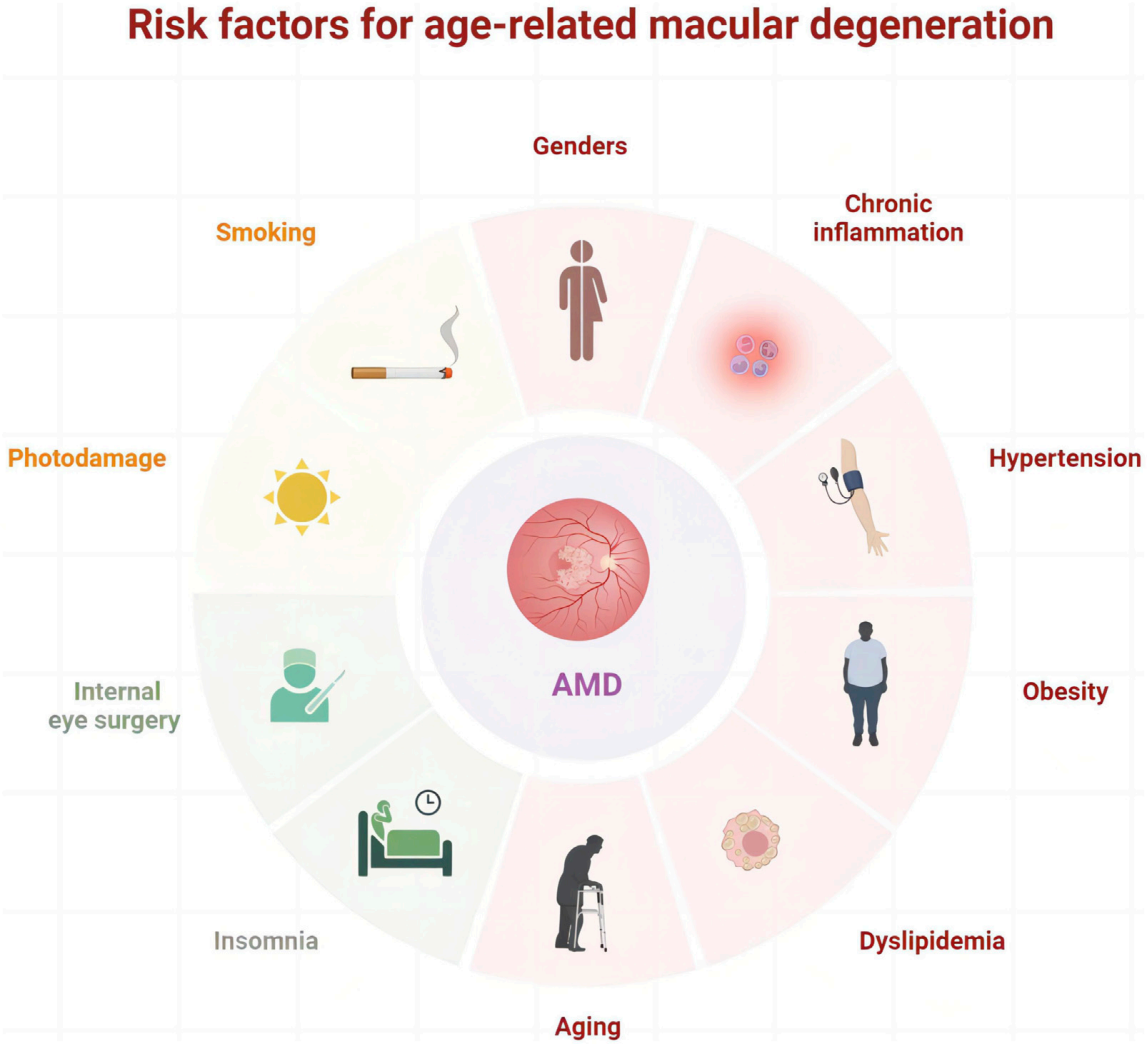
Risk factors associated with age-related macular degeneration.

Globally, age-related macular degeneration (AMD) is the third major factor contributing to substantial and irreversible vision impairment following cataracts and glaucoma. Advanced retinal pigment epithelial (RPE) atrophy or choroidal neovascularization can contribute to moderate to severe vision loss in patients with AMD (Ferris et al., 2013). The prevalence of AMD continues to rise worldwide and remarkably increases with age despite significant geographic and lifestyle differences. Currently, the global estimate of AMD prevalence is 196 million, projected to rise to 288 million by 2040 (Xiao et al., 2023), and is anticipated to exceed 498 million by 2050 (Lima-Fontes et al., 2022). The progression of AMD is significantly influenced by factors such as aging, photodamage, obesity (Tian et al., 2023), dyslipidemia, chronic inflammation (Ghosh et al., 2022; de Almeida Torres et al., 2022), hypertension (Zhang et al., 2022), smoking, and internal eye surgery (Ng Yin Ling et al., 2021; Abusharkh et al., 2023; Muraleva and Kolosova, 2023) (Figure 1). In Western medicine, treatments such as anti-vascular endothelial growth factor (anti-VEGF) (Al-Zamil and Yassin, 2017; Williamson et al., 2023), corticosteroids (Kaya et al., 2019), antioxidants (Evans and Lawrenson, 2017; Kushwah et al., 2023), laser therapy (Cohn et al., 2021), and photodynamic therapy (Ando et al., 2023) are utilized. Although these approaches can relieve symptoms, they cannot inhibit the development of AMD, and the treatment options for non-neovascular AMD are particularly limited.

**FIGURE 2:**
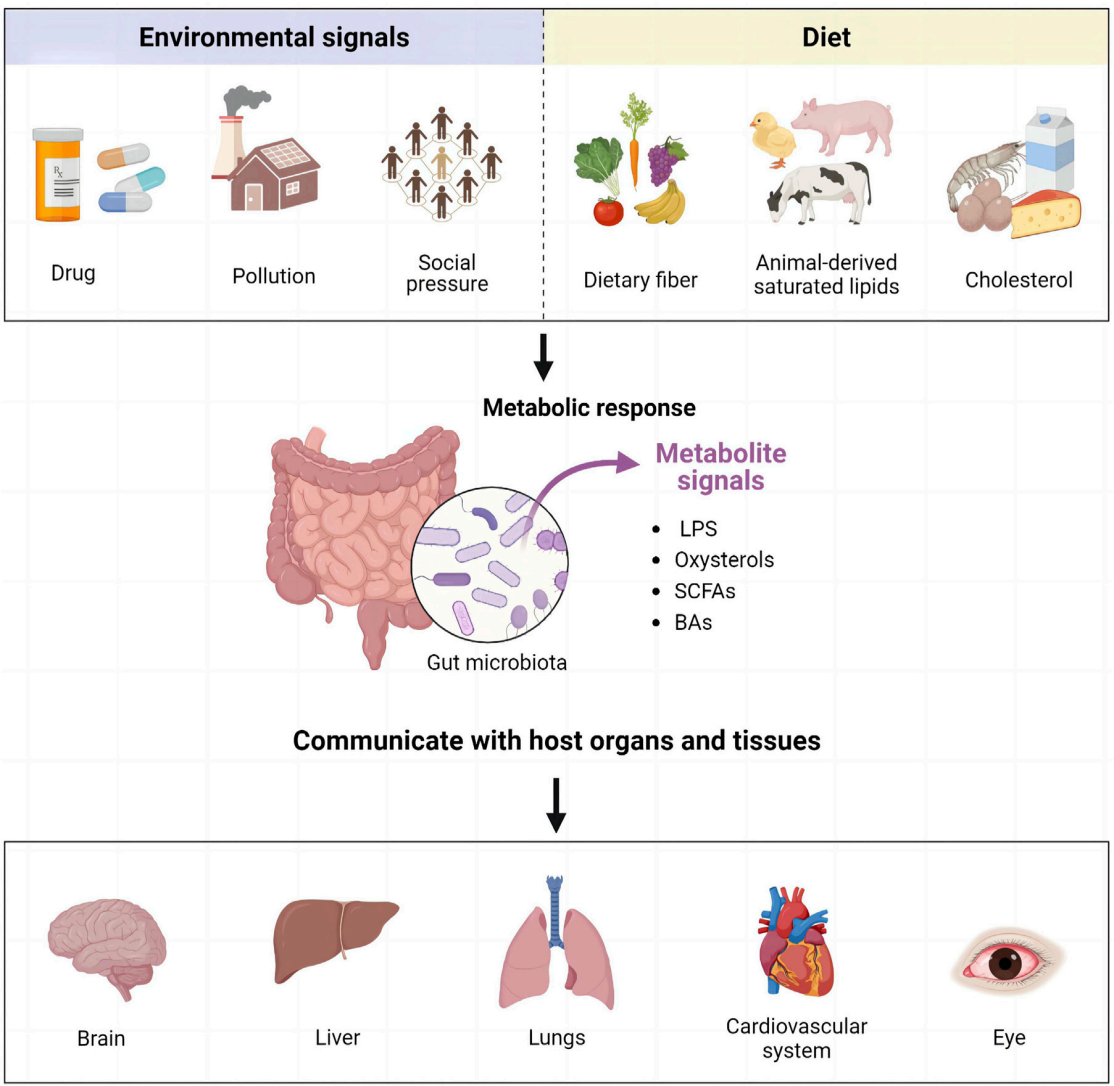
Representation of the “gut-organ axis.”

Gut microbiota (GM) coexists with the human body, playing an important role in maintaining the homeostasis of the internal environment and influencing various physiological functions. These functions include metabolism, synthesis of vitamins and other nutrients, antitoxicity, intestinal defense, regulation of immune organ development and maturation, and hematopoiesis (Afzaal et al., 2022; Ahlawat et al., 2021; Saxami et al., 2023). GM colonizes the gastrointestinal tract, influences the intestinal environment, and engages in bi-directional interactions with other organs (Ahlawat et al., 2021). This interaction has given rise to the concept of the “gut-organ axis,” which includes gut-brain, gut-liver, gut-lung, gut-cardiac, and gut-ocular axes (Guo et al., 2023a) (Figure 2). GM can convert environmental signals and dietary molecules into metabolite signals, facilitating communication with various organs and tissues of the host via various signaling pathways.

Recently, considerable evidence has accumulated suggesting a correlation between GM and AMD and its associated risks (Liu et al., 2023; Luo and Skondra, 2023; Mao et al., 2023). Regulating the GM is a potential therapeutic strategy for preventing AMD. Moreover, given its multi-component, multi-pathway, multi-target, multi-efficacy nature and low side effects, traditional Chinese medicine (TCM) has been used to manage similar fundus lesions since at least 620 CE (Cao et al., 2022). Recent research (Li et al., 2022a) has revealed that TCM is efficacious in treating AMD by modulating the GM, offering a novel approach to preventing and managing AMD. This review seeks to present the existing association between GM and AMD, evaluate the influence of TCM on GM, and explore the relationship of TCM and GM with AMD, aiming to provide new insights into treating AMD with TCM.

## 2 GM composition and metabolites influence AMD development

The GM is an intricate environment consisting of numerous microorganisms, such as viruses, bacteria, archaea, fungi, and protozoans, including up to 1,000 bacterial species. Healthy adults have up to 1×10^14^ bacteria in their intestines (Wang and Sun, 2022). Approximately 3.3 million genes exist in the human GM, corresponding to approximately 150 times the number of genes in the human genome (Rinninella et al., 2018). The GM includes the phyla *Firmicutes*, *Fusobacteria*, *Actinobacteria*, *Bacteroidetes*, *Verrucomicrobia*, and *Proteobacteria*. Among these, *Firmicutes* and *Bacteroidetes* are the most prevalent GM phyla, and the *Firmicutes/Bacteroidetes (F/B)* ratio is an important measure of microbial equilibrium in the whole gut.

The GM components are classified as probiotic, neutrophilic, or pathogenic based on their pathogenicity. The appropriate balance between probiotic and pathogenic bacteria can safeguard the intestinal mucosal barrier, promote digestion and absorption of nutrients, enhance immunity, and impede the invasion of pathogenic microorganisms into the body.

GM can further impact human health via the metabolites they release, such as lipopolysaccharides (LPS) (He et al., 2023a), oxysterols (Lefort and Cani, 2021), short-chain fatty acids (SCFAs), and bile acids (BAs) (Enriquez et al., 2023; Xiang et al., 2023). These metabolites function as signaling molecules that govern metabolic, immune, and inflammatory reactions in individuals with AMD.

### 2.1 GM composition influences AMD development

The occurrence and progression of AMD are linked to abnormal alterations in the GM composition. Although the relationship is unclear, GM dysregulation may play a role in AMD progression by enhancing the signaling pathway to activate the complement system abnormally (Xue et al., 2023). When the complement system is over-activated or dysregulated, the out-of-control complement system becomes a key link in triggering infection and inflammation. Enteric pathogens can break through the intestinal mucosal barrier, enter the eye via the somatic circulation, and trigger a localized inflammatory reaction, subsequently leading to a rise in the generation of interleukin (IL)-6, tumor necrosis factor-alpha (TNF-α), and vascular endothelial growth factor (VEGF-A). These cytokines were linked to the development of neovascular AMD (Zinkernagel et al., 2017). Investigation of the intestinal flora of patients with AMD using metagenomic sequencing revealed that the AMD group exhibited a notably diminished presence of *Firmicutes* and an elevated prevalence of *Proteobacteria* and *Bacteroidetes* compared with the control group. Moreover, the AMD group displayed a significant increase in *Escherichia-Shigella* at the genus level compared with that seen in the control group, whereas the proportions of *Blautia* and *Anaerostipes* were lower (Zhang et al., 2023).

Additionally, changes in the GM composition have been linked to oxidative stress, activation of the complement system, inflammation, and altered choroidal hemodynamics. Zinkernagel et al. (2017) conducted sequencing of intestinal macro-genomes in patients with AMD and healthy controls. They observed a higher abundance of *Anaerotruncus*, *Oscillibacter*, *Ruminococcus torques*, and *Eubacterium ventriosum* among individuals with AMD. In mice, these organisms are linked to the activation of proinflammatory chemokines, whereas in humans, they are associated with increased IL-6 and IL-8 levels. In a clinical case-control study (involving 85 patients with advanced AMD and 49 healthy individuals), Lin et al. discovered dysregulation of intestinal ecology that resulted in an increased abundance of *Holdemanella*, *Prevotella*, *Desulfovibrio*, and other bacteria. However, compared to that seen in the control group, *Oscillospira*, *Dorea*, and *Blautia* were reduced in abundance, indicating a correlation between certain bacteria and oxidative stress, inflammation, and heightened intestinal permeability in patients with AMD (Lin et al., 2021). Zysset-Burri et al. (2020) identified *Negativicutes* as a potential biomarker of neovascular AMD and found single nucleotide polymorphisms in the recombinant human complement factor H gene linked to AMD. *Negativicutes* correlated positively with the AMD-associated complement factor H risk allele.

GM-based studies conducted in mouse models have demonstrated a correlation between *Clostridials* and *Firmicutes* with retinal damage, whereas *Bacteroidales* have been associated with AMD repair. A positive correlation was observed between the relative abundance of *Clostridials* and *Firmicutes* unknown species-level genome bins and lesion size, central pit thickness, hemorrhage size, and angiographic leakage. In contrast, a negative correlation was observed between lesion and hemorrhage size and the relative abundance of *Bacteroidales* (Xue et al., 2023).

In addition, alterations in the GM may impact the metabolic risk factors for AMD. *Firmicutes* may promote obesity and chronic inflammation. The elevated abundance of *Firmicute*s following the intake of high-fat foods allows greater energy acquisition, leading to weight gain and obesity (Ley et al., 2006). Feeding mice a high-fat diet (HFD) can contribute to obesity, chronic low-grade inflammation, increased intestinal permeability, and ultimately worsen choroidal neovascularization by increasing the abundance of *Firmicutes* (Xiao et al., 2022). Moreover, *Lactobacillus acidophilus* is negatively associated with obesity. By reducing the *F/B* ratio and controlling the expression of genes associated with lipolysis, lipid synthesis, and energy metabolism, this bacteria effectively counteracts HFD-induced dysbiosis of intestinal ecology and upholds the integrity of the intestinal barrier (Kang et al., 2022).

Additionally, a decrease in the *F/B* ratio signals senescence. Compared to healthy young adults, who have a high proportion of *Firmicutes*, older adults demonstrate a decrease in *Bifidobacteria* and an increase in *Bacteroidetes*, as well as a decline in gut microbial diversity, with a reduction in the relative abundance of core species and an augment in the colonization of opportunistic species (*Betaproteobacteria*) (Biagi et al., 2017). *Bacteroidetes* and *Firmicutes* are the most common bacteria found in the GM of older individuals (Wu et al., 2021). Older individuals have a high percentage of *Bacteroidetes* and an increased relative abundance of *Clostridium* in their GM compared to younger individuals, and there is also a reduced abundance of *Ruminococcus torque* and *Prevotella* in this age group. *Bacteroidetes* constitute the majority (53%) of the primary microbiota in older individuals, in contrast to the reduced level (8%–27%) present in healthy young individuals.

*Bifidobacteria* are believed to reduce inflammatory responses in older individuals. A randomized controlled double-blind crossover trial (Macfarlane et al., 2013) showed that combined administration of *Bifidobacteria* and the prebiotic inulin led to an increase in *Bifidobacteria* and a reduction in *Proteobacteria* in the host. This regimen also increased butyrate production and significantly decreased TNF-α, a proinflammatory cytokine, in the peripheral blood.

GM dysbiosis is an imbalance between probiotics and pathogenic bacteria. The composition and function of intestinal microorganisms change with host genes, diet, internal environment, and other factors. When the equilibrium of the intestinal microecology is disturbed, the number of foreign microorganisms and pathogenic bacteria in the body increases, inducing intestinal metabolic disorders and immune system dysfunction, leading to morbidity. The body can mobilize more probiotics to restore a balanced and healthy microecological environment to combat these pathogens.

### 2.2 GM metabolites influence AMD development

#### 2.2.1 Lipopolysaccharides influence AMD development

The outer membrane of Gram-negative bacteria contains LPS, which consist of a hydrophobic domain-endotoxin-lipid A, a hydrophilic O-antigen, and a central polysaccharide (Brodzikowska et al., 2022). Two molecules of 3-hydroxy fatty acids are esterified to the glucosamine backbone of lipid A, and another two are connected via an amide linkage. Lipid A serves as the pathogen-associated molecular pattern of LPS. Increased LPS concentrations act as potent immune activators and toll-like receptor (TLR) 4 (TLR4) ligands, triggering inflammation (Chassaing and Gewirtz, 2014; Scott et al., 2017). Within the eye, various cells, such as perivascular macrophages, microglia, photoreceptors, dendritic cells, and RPE cells, exhibit proinflammatory signals via LPS (Tsioti et al., 2022). LPS-induced retinal explants exhibited a notable neuroinflammatory reaction, marked by deterioration of neurons and increased levels of various cytokines (Ghosh et al., 2018).

Furthermore, emerging evidence strongly suggests that low-grade inflammation caused by LPS plays a role in the progression of AMD. Larsen et al. (2023) observed increased levels of esterified 3-hydroxy fatty acids (indicative of LPS burden) in blood samples, indicating a potential role of LPS exposure in the early stages of AMD pathophysiology. The above suggests that LPS leads to a range of acute and chronic inflammatory responses, generating edema, exudation, and neovascularization, further affecting the development and progression of AMD (Ibbett et al., 2014).

Researchers have explained the risk of LPS-induced AMD via various mechanisms, such as immune inflammation, peroxidative damage, and RPE senescence. Liu et al. (2019) proposed that LPS promotes the production of inflammatory cytokines and apoptosis of RPE cells by inducing miR-21-3p expression, which can lead to AMD. LPS-induced miR-21-3p overexpression increased the protein and mRNA levels of the inflammatory cytokines monocyte chemoattractant protein-1 (MCP-1) and IL-6 in RPE cells while elevating apoptosis, caspase-3 activity, and levels of cleaved caspase-3 and poly-(ADP-ribose) polymerase proteins, which exacerbate inflammatory responses and apoptosis. LPS not only stimulates the phosphorylation of extracellular signal-regulated kinase (ERK1/2) and nuclear factor kappa-B (NF-κB), but also strongly increases the expression levels of IL-1β, IL-6, IL-12, VEGF, TNF, and TNF-related apoptosis-inducing ligand. Furthermore, exposure to LPS increases the expression of glutathione peroxidase and mitochondrial manganese superoxide dismutase, leading to oxidative stress in the cells. However, Ozal et al. (2018) found that esculetin downregulates the secretion of proinflammatory cytokines and decreases NF-κB activation, ERK1/2 phosphorylation, and VEGF levels, consequently suppressing oxidative stress and inflammation, protecting against LPS-mediated RPE cell death, and preventing AMD development.

LPS production by Gram-negative bacteria in the intestinal flora is involved in HFD-induced obesity. Gram-negative bacteria secrete LPS, which binds to the complex receptor CD4/TLR4 on the surface of immune cells, releasing proinflammatory cytokines and developing inflammatory responses and metabolic disorders, ultimately leading to diseases such as obesity (Davis, 2016). Therefore, therapeutic options that balance LPS levels are of interest in AMD.

#### 2.2.2 Oxysterols influence AMD development

Oxysterols are cholesterol derivatives produced via enzymatic or free radical oxidation associated with oxidative stress, inflammation, and apoptosis (Testa et al., 2018; Zarrouk et al., 2020; de Medina et al., 2022). Several studies have suggested a correlation between retinal degeneration and cholesterol metabolism, specifically the transformation of cholesterol into oxysterols and the degeneration of the retina (Pfeffer et al., 2021; Zhang et al., 2021). Dasari et al. (2011) observed that providing rabbits with a diet high in cholesterol for 12 weeks resulted in heightened β-amyloid (Aβ) levels in the retina, oxidative harm, apoptosis, and elevated accumulation of cholesterol and oxysterols (24-hydroxycholesterol (24-OH), 27-hydroxycholesterol (27-OH), 4β-hydroxycholesterol, 7α-hydroxycholesterol, 25-hydroxycholesterol (25-OH), 7-ketocholesterol (7KC), and 7β-hydroxycholesterol (7β-OH)). A prior study (Dasari et al., 2010) revealed that 27-OH induced toxicity in RPE cells via the stimulation of Aβ1–42 peptide production, elevation of stress markers specific to the endoplasmic reticulum (cysteinyl asparaginase-12 and C/EBP homologous protein), reduction in dysregulation of Ca^2+^ homeostasis, mitochondrial membrane potential, oxidative stress (as indicated by glutathione depletion and reactive oxygen species (ROS) production), and apoptosis. Studies have demonstrated that cholesterol metabolism and its oxidation byproducts, including 7KC, can adversely affect RPE cells, as 7KC triggers oxidative stress and cell death. Various alterations have been noted in RPE cells in the presence of 7KC, such as damage to mtDNA, mitochondrial dysfunction, increased production of ROS/reactive nitrogen species (Gramajo et al., 2010), and activation of caspase-8, -12, and -3 (Neekhra et al., 2007). 7β-OH induces a caspase-3-independent mode of cell death related to lysosomal destabilization, which plays a significant role in the signaling pathways resulting in cell death (Malvitte et al., 2008). These findings collectively demonstrate the association between oxysterols and AMD.

Furthermore, oxysterols cause a rise in inflammatory cytokines in retinal cells. The presence of 25-OH (20–30 μg/mL) in human retinal pigment epithelium (ARPE-19) cells triggered the release of IL-8 (via the MEK/ERK1/2 pathway), VEGF, and MCP-1. The administration of 25-OH upregulated IL-8 transcription and secretion, facilitated by ERK1/2 and phosphoinositol-3 kinase activities and the involvement of transcription factor activator protein 1 and NF-κB. The presence of 7KC and 7β-OH elevated the secretion of IL-1β and IL-6 in ARPE-19 cells (Dugas et al., 2010). Huang et al. (2012) also demonstrated that 7KC (8 μM) enhanced levels of TNF-α, IL-1β, IL-6, IL-8, transforming growth factor beta 1 (TGF-β1), and VEGF by a mechanism that may involve endoplasmic reticulum stress. Larrayoz et al. (2010) found that 7KC induced cytokine production via the kinase signaling pathways ERK, p38MAPK, and AKT-PKCζ-NF-κB via interactions in the plasma membrane. The activation of NF-κB was linked to the MAPK/ERK pathway. These findings illustrate that oxysterols, particularly 25-OH, 7βOH, and 7KC, can trigger oxidative stress, apoptosis, and inflammation in RPE cells, thereby inducing retinal degeneration. Therefore, lowering oxysterol levels may be beneficial in preventing and treating AMD.

#### 2.2.3 Other GM metabolites influence AMD development

Organic fatty acids with a carbon chain length of less than six, known as short-chain fatty acids (SCFAs), are generated by bacteria in the gut, including 41 families, such as Lactobacillaceae*,* Clostridiaceae*,* Christensenellaceae Bifidobacteriaceae*,* Lachnospiraceae*,* and *Akkermansia muciniphila*. The main components of SCFAs are acetate, propionate, and butyrate. Acetate, constituting 50%–60% of SCFAs, is produced by *Bifidobacteria* and *Lactobacilli*, along with bacteria such as *Clostridium* spp., *Anaerotruncus*, *Lachnospira*, *Akkermansia Muciniphila*, and *Streptococcus* spp. The *Bacteroidetes* and Negativicutes class of *Firmicutes* synthesize propionate via a succinate route, utilizing vitamin B12 to transform succinate into propionate. Other Negativicutes bacteria form propionate from lactate via the succinate, such as *Veillonella* spp. or acrylate pathways, such as *Coprococcus catus*, Lachnospiraceae, and *Megasphaera elsdenii*. *Eubacterium rectale* and *Roseburia* spp., members of the *Clostridium coccoides* group (clostridial cluster XIVa), along with *Faecalibacterium prausnitzii*, a member of the *Clostridium leptum* group (clostridial cluster IV), are capable of producing butyrate. Besides the two prevalent human clusters, clostridial clusters I, III, XV, and XVI can also produce butyrate. SCFAs enter the bloodstream via the intestine, directly influencing the functioning of peripheral tissues and metabolic processes. SCFAs are crucial in regulating gene expression, controlling host metabolic processes (cell proliferation, diversification, and apoptosis), inflammatory reactions, and energy supply (Abdalkareem Jasim et al., 2022; Fillier et al., 2022; Rekha et al., 2022; Anachad et al., 2023; Ney et al., 2023). The metabolic responses mediated by SCFA receptors also influence obesity, contributing to AMD (Tian et al., 2023). Propionic acid increases peptide YY and glucagon-like peptide 1 (GLP-1) production and secretion, which helps to control obesity. Propionate and butyrate have the potential to combat obesity by stimulating intestinal gluconeogenesis, thereby enhancing metabolic wellbeing. Butyric acid upregulates the lipocalin-mediated AMP-activated protein kinase (AMPK) pathway, promoting mitochondrial biosynthesis and fatty acid oxidation by inhibiting histone deacetylases and augmenting peroxisome proliferator-activated receptor alpha (PPAR-α). SCFAs further ameliorate obesity by decreasing PPAR-α expression, increasing adipose tissue metabolism, and reducing body fat accumulation (Anachad et al., 2023).

Additionally, SCFAs may exert anti-inflammatory effects. SCFAs can traverse the blood–ocular barrier via the circulatory system. Chen et al. (2021) demonstrated the ability of high-dose, intraperitoneally administered SCFAs to reach the eye and impede LPS-induced endophthalmitis. SCFAs hinder TNF-α, IL-6, and the chemokines C-X-C motif chemokine ligand 1 and C-X-C motif chemokine ligand 12 when exposed to inflammatory stimuli *in vitro*, including ligands for TLR and IL-17. SCFAs reduce inflammatory mediators produced by LPS-stimulated retinal astrocytes and enhance the ability of retinal astrocytes to activate T cells, thereby treating AMD. Growing evidence indicates that SCFAs help to improve AMD and its associated risk factors. However, further clinical trials and animal experiments are required to validate these findings.

Bile acids (BAs) are synthesized from cholesterol within hepatocytes. The gallbladder stores BAs, which are then released into the intestine, aiding in the absorption of dietary fats and vitamins. Research indicates that GM significantly influences BA metabolism (Wahlström et al., 2016). Primary BAs—cholic acid and chenodeoxycholic acid—are produced from liver cholesterol, whereas GM generates secondary BAs via hydroxylation, deconjugation, epimerization, or oxidation processes. Both primary and secondary BAs in the liver are typically linked to glycine or taurine. Research has demonstrated that BAs positively impact experimental models studying retinal disorders. Warden et al. (2020) found that tauroursodeoxycholic acid promotes the phagocytosis of outer segments of photoreceptor cells by RPE cells, inhibits human retinal endothelial cell proliferation *in vitro*, and inhibits choroidal neovascularization. The BA taurocholic acid shields HRPEpiC primary retinal epithelial cells from oxidative stress-induced damage to their structure and function while also inhibiting VEGF-induced angiogenesis in choroidal endothelial cells. Moreover, glycine-bound BAs protect RPE cells from oxidative harm and impede VEGF-triggered angiogenesis in choroidal endothelial cells. Glycine-bound BAs offer protection against both atrophic and neovascular AMD.

**FIGURE 3:**
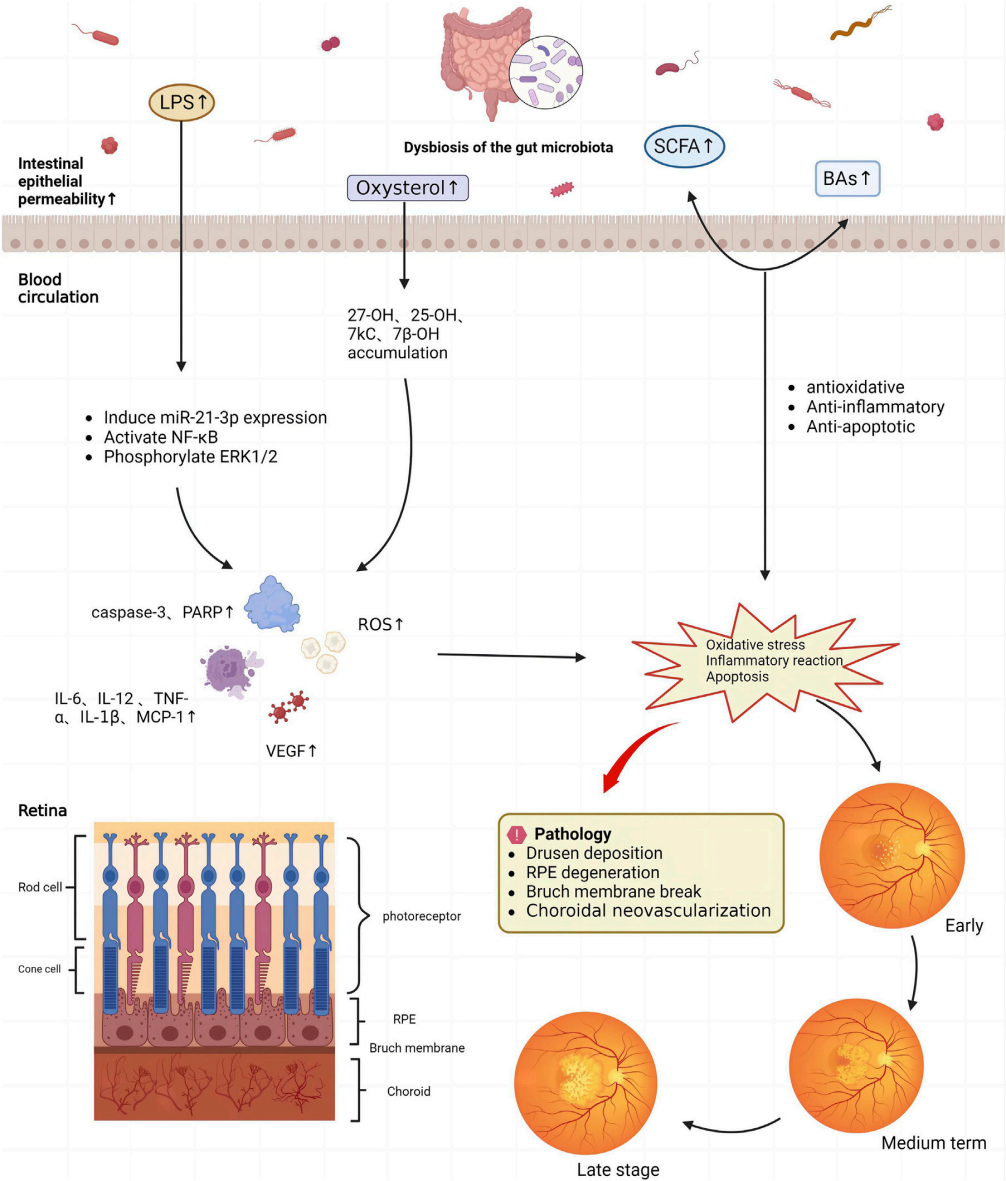
Mechanism underlying the effects of gut microbiota metabolites on age-related macular degeneration.

In summary, LPS and oxysterols can cause oxidative stress, cellular senescence, and various acute and chronic inflammatory responses that further affect the occurrence and development of AMD (Figure 3). SCFAs improve AMD symptoms by regulating cell proliferation, differentiation, and apoptosis, inhibiting inflammatory responses, and providing energy. BAs improve lipid metabolism, protect oxidative stress-induced RPE cells, and inhibit choroidal neoangiogenesis. Consequently, the progression of AMD can be significantly slowed and postponed via the regulation of intestinal flora metabolites, including LPS balance, oxysterol reduction, and an increase in SCFAs and BAs (Guo et al., 2023b).

## 3 Mechanisms by which the GM plays a role in TCM on AMD

The spleen and stomach are important organs in the digestive system of the human body, with the functions of digesting and absorbing food and maintaining normal metabolism and energy supply. TCM theory suggests that the spleen and stomach have physiological functions analogous to those of the GM. After preliminary digestion by the stomach, filtering, and absorption by the small intestine, food is transformed into subtle substances, that is, qi and blood, by the spleen. These substances are subsequently circulated throughout the body to sustain the regular physiological functions of other organs and tissues. Proper spleen and stomach functioning facilitates sustained energy metabolism, and improves immunity against pathogen invasion (Wu et al., 2020; Ye et al., 2022). Studies have shown that when the “transformation” and “defense” functions of the spleen and stomach are normal, they are similar to the role of the intestinal flora on the body’s metabolism and immune function. When the intestinal flora maintains homeostasis, the body’s nutrient metabolism functions normally, and the spleen and stomach transform food into nutrients. In contrast, when the intestinal microecology is disturbed, the host is susceptible to pathogens, and the spleen and stomach malfunction, leading to qi and blood deficiencies, as well as poisonous metabolites.

The defining feature of AMD is the degeneration of the macular region. According to the principles of TCM, the spleen and stomach are intricately linked to this disease, and AMD is characterized by weakness in the spleen and stomach. Food preferences, improper rest, stress, anxiety, and old age can lead to malfunction of the spleen and stomach, making it difficult to generate qi and blood. Weak qi and blood impede nutrient supply to the eye tissues, fostering disease. The poisonous metabolites produced accumulate in tissue cells over time and the functional structure of the retina is impaired (Yang and Jia, 2013; Li, 2022).

The metabolism of the intestinal flora is intricately linked to the functions of the spleen and stomach. LPS and oxysterols are biomarkers of poisonous metabolites in TCM. The GM converts LPS and oxysterols from food and is associated with oxidative stress, various acute and chronic inflammatory responses, and apoptosis. Dysbiosis of the microbiota and an increase in pathogenic bacteria can be regarded as “external toxins.” In addition, the dysfunction of microbiota-derived metabolites can be seen as “internal toxins,” leading to inflammation, oxidative stress, dyslipidemia, obesity, aging, and VEGF production. This is also the pathological basis of AMD, which explains the poisonous metabolites of TCM from a modern microbiological perspective. Furthermore, GM metabolites, such as SCFAs and BAs, can protect the retinal pigment epithelium from oxidative damage, inflammatory stimuli, and apoptosis, which can have a protective effect against retinal damage, such as light-induced retinopathy and AMD. This suggests that TCM can be used to intervene in AMD by adjusting the intestinal flora, increasing SCFAs and BAs, and ameliorating oxidative damage, apoptosis, inflammation, and altered choroidal hemodynamics.

## 4 Therapeutic intervention for AMD with TCM

Most botanical drugs are orally ingested and absorbed through the digestive system. The components of these botanical drugs are metabolized or transformed by intestinal microorganisms, producing new biologically active molecules that facilitate drug absorption into the bloodstream. The composition and structure of the GM are affected by TCM metabolites, which in turn affect the function of diseased organs and tissues via the action of the intestinal flora. Research has indicated that a single botanical drug’s components and metabolites may improve AMD symptoms and risk factors by modulating intestinal microorganisms. Below, we review therapeutic interventions using botanical drugs based on categorizing natural chemical components, single botanical drugs, and botanical drug decoctions.

### 4.1 Natural chemical components

#### 4.1.1 Flavonoids

Flavonoids are compounds that are abundantly found in plants (Table 1). They have different phenolic structures consisting of two benzene rings with phenolic hydroxyl groups, primarily in glycosidic and free forms. These compounds exhibit antioxidative, anti-inflammatory (Guo et al., 2023c), antimicrobial, anti-apoptotic, neovascularization inhibiting, and central nervous and cardiovascular system protective effects (Lan et al., 2023a). Typically, flavonoid glycosides contain glucose bonds. Glucosides with water-soluble sugar components have low pharmacological activity and are difficult to absorb in the intestine, leading to a lack of bioavailability. Nevertheless, enzymatic degradation, hydrolysis, reduction, dehydroxylation, and other reactions involving intestinal microbiota can convert most flavonoids into simple phenolic acids, leading to their absorption and subsequent bioavailability enhancement.

**TABLE 1:** Natural chemical components and gut microbiota.

Natural chemical components	Physiological function related to gut microbiota	Gut microbiota	References
Flavonoids	(1) Regulate the number and abundance of gut microbiota	Increased: Bifidobacterium Decreased: Bilophila, Lachnoclostridium, and Haemophilus, Bacteroides and Clostridium pullulans	Pan et al. (2023), Odamaki et al. (2016), Sun et al. (2023), Carrera-Quintanar et al. (2018)
	(2) Regulate inflammatory factor gene expression and inhibit inflammatory responses		
	(3) Reduce ROS production, alter AKT/GSK-3β and NrF2/ARE signaling pathways, and regulate antioxidant gene expression		
	(4) Inhibit adipogenesis to regulate obesity		
Berberine	(1) Inhibits the LPS/NF-κB signaling pathway to improve the metabolic and inflammatory state of organisms	Increased: Ruminococcus, Desulfovibrio vulnificus, Lactobacillus, and Akkermansia muciniphila Decreased: *Aspergillus*, *Trematode spirochetes*, and the ratio of *Firmicutes* to *Bacteroidetes*	Zhang et al. (2012), Li et al. (2018), Liao et al. (2020), Shen et al. (2021), Yang et al. (2022)
	(2) Activates AMPK pathway to combat oxidative stress		
	(3) Promotes the production of SCFAs		
	(4) Corrects the imbalance of the GM ratio		
Resveratrol	(1) Promotes the metabolism of SCFAs and increases the level of butyric acid	Increased: *Bacillus anthropophilus, Lactobacillus, Bifidobacterium*, and the ratio of *Firmicutes* to *Bacteroidetes *Decreased: *Firmicutes* and *Enterococcus faecalis*	Qiao et al. (2014), Zhang et al. (2015), Chen et al. (2016), Maugeri et al. (2018), Alrafas et al. (2019), Dull et al. (2019), Li et al. (2022b)
	(2) Anti-inflammatory and anti-metabolic disorder effects through various pathways, such as NF-κB, arachidonic acid, AP-1, or AHR		
	(3) Reduces the extent of oxidative stress damage in RPE cells		
	(4) Activates SIRT1 and downregulates HIF-1α expression and VEGF secretion in RPE cells		
	(5) Improves hyperlipidemia		

Flavonoids can be divided into two primary groups depending on how they control the GM. Some flavonoids can be metabolized by the GM and act as substrates for various catalytic reactions involving various enzyme systems produced by the GM. Consequently, the bacteria responsible for these reactions tend to propagate (Feng et al., 2018). In contrast, some flavonoids influence the cell membranes of specific bacteria (such as *Staphylococcus aureus* and *Escherichia coli*), either by directly disrupting the lipid bilayer of the cell membrane or by modifying the permeability of the cell membrane, ultimately hindering the proliferation of these bacteria (Xie et al., 2015). Flavonoids modulate the relative abundance of probiotic and harmful bacteria. Pan et al. (2023) found that certain flavonoids (particularly hesperidin-7-O-glucoside, prunin, and isoquercitrin) possess bactericidal, antiviral, and anti-inflammatory properties.

Moreover, flavonoids also modulate the abundance and number of intestinal microbiota, leading to a considerable reduction in total SCFA production. By using *in vitro* simulated fermentation technology to study the fecal microbial composition of healthy individuals, it has been demonstrated that hesperidin-7-O-glucoside, prunin, and isoquercetin increase *Bifidobacterium* and result in a decrease in the relative abundance of *Bilophila, Lachnoclostridium*, promoting intestinal mucosal absorption and digestion, biobarrier function (Odamaki et al., 2016), and immunomodulation (Milani et al., 2017). Flavonoids reduce the effects of harmful bacteria. Hesperidin-7-O-glucoside, naringenin, and lisin all significantly reduce the levels of *Haemophilus* and the relative abundance of harmful bacteria, such as *Clostridium pullulans* and *Bacteroides*, suggesting that flavonoids can regulate signaling pathways, such as TLR4/NF-κB, NOD-like receptor family pyrin domain containing 3, and MAPK, along with the expression of the genes encoding matrix metalloproteinase-9, IL-1β, IL-6, IL-8, soluble intercellular adhesion molecule-1, MCP-1, and other inflammatory factors, suppressing the inflammatory response (Sun et al., 2023). The presence of flavonols, a type of flavonoid, facilitates the proliferation of beneficial bacteria, such as *Lactobacillus* and *Bifidobacterium*, while diminishing the presence of *Clostridium* spp. Taken together, hesperidin-7-O-glucoside, lisinin, and isoquercitrin, which are flavonoid monoglycosides containing only one glucose group in their structures, can lead to an increase in beneficial intestinal bacteria and a decline in harmful intestinal bacteria. This action helps ameliorate intestinal dysbiosis and diminish the impact of other factors on the intestinal flora.

Natural flavonoid products also have antioxidant properties that reduce oxidative stress and inhibit the activation of various signaling pathways to ameliorate inflammatory diseases. By improving the dysregulation of intestinal dynamic homeostasis *in vivo*, ROS production is reduced, the AKT/glycogen synthase kinase-3-beta (GSK-3β) and Nuclear factor erythroid-derived-2-like 2 (Nrf2)/antioxidant response element (ARE) signaling pathways are directly altered, and the expression of antioxidant genes, such as *NAD*(*P*)*H quinone oxidoreductase 1, heme oxygenase-1, glutamate-cysteine ligase modifier, glutathione*, and *superoxide dismutase* (*SOD*), are regulated (Sun et al., 2023).

Increasingly, flavonoid metabolites, which are biologically active compounds, play a role in influencing the gut microbiome and reducing signs of obesity by hindering adipogenesis. Flavonoids, with their anti-inflammatory and antioxidant properties, impede the production of ROS and the cyclooxygenase (COX)-2 and NF-κB signaling pathways, thereby influencing obesity development and inflammatory responses (Carrera-Quintanar et al., 2018). Detaram et al. (2021) suggested that intake of higher levels of certain flavonoids may improve vision outcomes in patients with AMD. These findings were consistent with those of the Blue Mountains Eye Study, which revealed a connection between the overall consumption of flavonoids and reduced probability of AMD.

These findings suggest that flavonoids help control the development of AMD by modulating the gut microbial composition to slow inflammation, oxidative stress, dyslipidemia, and obesity.

#### 4.1.2 Berberine

Berberine (BBR) or BBR hydrochloride is an isoquinoline alkaloid derived from *Coptis chinensis* Franch. [Ranunculaceae; coptidis rhizoma]. It exhibits pharmacological properties, including anti-inflammatory, cardioprotective, and hypoglycemic effects (Miao and Cui, 2022; Saha et al., 2023). Intestinal flora enhances the bioavailability of orally administered BBR. The intestinal microbiota transforms BBR into absorbable dihydroberberine, which is five times more absorbable than BBR. Dihydroberberine is not stable in solution and can be reoxidized to BBR in intestinal tissues (Cheng et al., 2021).

BBR may inhibit inflammatory responses, oxidative stress, and apoptosis by altering the growth of various intestinal bacteria. It can reduce pathogenic bacteria and improve the metabolic and inflammatory state of organisms by inhibiting the LPS/NF-κB signaling pathway to promote the production of SCFAs, improving the relative abundance of GM, augmenting the relative abundance of SCFA-producing flora, and diminishing endotoxins (Shen et al., 2021). Yang et al. (2022) found that BBR modulates the GM and inhibits the initiation of the TLR4 signaling pathway, as well as the release of the nucleotide-binding oligomerization domain-like receptor protein 3 inflammasome and its cytokines. Li et al. (2018) also found that BBR inhibits oxidative damage caused by hydrogen peroxide (H_2_O_2_) in the human D407 RPE cell line. Pretreatment of D407 cells with BBR effectively inhibited apoptosis caused by H_2_O_2_ by rectifying irregular alterations in the nuclear structure, impeding the reduction in mitochondrial membrane potential, decreasing lactate dehydrogenase release, and impeding the activity of caspase 3/7 induced by H_2_O_2_. Western blot analysis revealed that BBR induced the phosphorylation and activation of AMPK in D407 cells in a manner that is dependent on the amount of time and dosage administered. In contrast, the action of BBR was inhibited by treating cells with compound C or reducing AMPK levels with specific siRNAs. Primary cultured human RPE cells yielded comparable outcomes. The collective findings indicate that BBR can protect RPE cells against oxidative stress via AMPK pathway activation, thereby addressing and slowing the progression of AMD.

Moreover, BBR can correct dyslipidemia by regulating the GM to improve AMD. BBR can boost the presence of *Ruminococcus, Desulfovibrio vulnificus, Lactobacillus*, and *A. muciniphila*, decline pathogenic bacteria, such as *Aspergillus* and *Trematode spirochetes*, promote the production of intestinal microbial metabolites such as SCFAs, and restore the breakdown and assimilation of glycolysis, amino acid metabolism, and carbohydrates (Liao et al., 2020), rectifying the imbalance in the rat intestinal flora ratio caused by a HFD, reducing the proportion of *Firmicutes* to *Bacteroidetes*, safeguarding the intestinal mucosal barrier, improving intestinal permeability, and preventing hyperlipidemia (Zhang et al., 2012).

#### 4.1.3 Resveratrol

Resveratrol is a polyphenolic compound with a low molecular weight naturally found in grapes, berries, and mulberries. It is recognized as a natural anti-inflammatory agent with a broad spectrum of medicinal effects, including anti-inflammatory, antimicrobial, antioxidant, immune-modulatory, anti-cardiovascular, and hepatocyte-protective activities (Boldyreva et al., 2023; Ghavidel et al., 2023; Santos et al., 2023). However, its notably low bioavailability poses a challenge in explaining the material basis of its *in vivo* pharmacodynamic effects. Several recent studies have shown that the exertion of its pharmacodynamic effects is associated with intestinal flora. Regarding intestinal flora regulation, resveratrol reduces the number of *Bacteroidetes*, promotes the metabolism of SCFAs, increases the level of butyric acid, and restores the intestinal flora to a homeostatic level (Alrafas et al., 2019).

Resveratrol has the potential to regulate the abundance of GM, thereby exerting antioxidant, anti-inflammatory, and anti-VEGF properties. Dull et al. (2019) showed that resveratrol can regulate the composition of gut bacteria, such as raising the amount of *Bifidobacterium*, *Bacillus anthropophilus*, and *Lactobacillus* and decreasing the amount of *Enterococcus faecalis* and *Firmicutes*. It may also exert anti-metabolic and anti-inflammatory effects via various pathways, such as NF-κB, arachidonic acid, activator protein-1, or aryl hydrocarbon receptor (AHR), diminishing proinflammatory elements and moderate granulocyte infiltration, functioning as an anti-proliferative and anti-inflammatory agent (Li et al., 2022b). Simultaneously, resveratrol can diminish the amount of oxidative stress damage in RPE cells by scavenging excess ROS, enhancing antioxidant enzyme activity, improving mitochondrial function, agonizing PPAR-α and peroxisome proliferator-activated receptor-δ, and up-regulating the mRNA expression of antioxidant genes *BCL2* and *HO1*, thereby inhibiting the development of AMD. Maugeri et al. (2018) found that resveratrol restored the methylation levels of long-interspersed nuclear element-1 and reduced the oxidative stress and inflammatory response in ARPE-19 cells by regulating the functions of Sirtuin 1 and DNA methyltransferase. Resveratrol can also inhibit the phosphorylation and activation of vascular endothelial growth factor receptor 2 in endothelial cells and exert anti-AMD effects by activating SIRT1, as well as down-regulating the expression level of hypoxia-inducible factor-1α (HIF-1α) and VEGF secretion in RPE cells (Zhang et al., 2015).

Resveratrol can ameliorate intestinal flora dysbiosis caused by a HFD in mice, increase the amount of *Bacteroidetes* and *Firmicutes*, stimulate the propagation of *Lactobacillus* and *Bifidobacterium,* restore the imbalance in lipid metabolism, and significantly boost the expression of *FIAF* to reduce the number of blood lipids (Qiao et al., 2014). Chen et al. (2016) observed the impact of resveratrol on the GM and trimethylamine-N-oxide (TMAO) levels in apolipoprotein E knockout and choline-fed C57BL/6J mice. They found that plasma trimethylamine and TMAO levels decreased in the choline-fed resveratrol-treated group. Additionally, resveratrol intervention caused a decrease in *Firmicutes* and an increase in *Bacteroidetes* in the intestinal tracts of these mice, which indicated that resveratrol could reduce the abundance of detrimental bacteria, as well as effectively increase the relative abundance of advantageous microorganisms and the amount of intestinal pathogenic metabolites. These actions significantly enhance the hyperlipidemic state, restore the normal level of blood lipids, and could aid in preventing and controlling AMD by regulating its risk factors.

### 4.2 Single botanical drugs

#### 4.2.1 *Poria cocos* (Schw.) Wolf [*Polyporaceae*; Poria]

*Poria* is a common botanical drug (Table 2). Its main chemical components are polysaccharides, triterpenoids, sterols, and trace elements, such as calcium, iron, zinc, selenium, potassium, sodium, and phosphorus. *Poria* polysaccharides and triterpenoids have major pharmacological activities. Contemporary pharmacological research has demonstrated that *Poria* coccinea has antioxidant, immunomodulatory, anti-inflammatory, and intestinal microbiota-regulatory effects (Xu et al., 2023). *Poria* increases the beneficial bacteria *Lactobacillus* and *Bifidobacterium* and decreases *Vibrio desulfuricans*, inflammation-associated bacteria *Mucor* spp., and *Staphylococcus* to attenuate oxidative stress, inflammatory responses, and apoptosis (Ye et al., 2023). Huai et al. (2023) evaluated the unique ability of *Poria* to promote water metabolism in the body for the treatment of diabetic macular edema. They ranked it high among the therapeutic drugs for proliferative diabetic retinopathy.

**TABLE 2:** Single botanical drugs and gut microbiota.

Single botanical drugs	Active ingredients	Physiological function related to gut microbiota	Gut microbiota	References
Poria	Poria polysaccharides	Regulates intestinal bacterial communities	Increases the butyrate-producing biomass of *Clostridium perfringens*	Sun et al. (2020a)
		Promotes lipid metabolism		
		Reduces inflammatory response		
	Poria oligosaccharides	Restores intestinal homeostasis, modulates intestinal flora–host metabolite interactions to improve body metabolic homeostasis and inflammation	Modulates the abundance of Odoribacter, Muribaculum, Oscillibacter, *Escherichia coli*, and Turicibacter	Lan et al. (2023b)
		Restores imbalances in the gut microbiota	Increased: Lactobacillus and Clostridium difficile Decreased: Helicobacter, Lachnospiraceae family, Alistipes, Ruminococcus, Faecalibacterium, Desulfovibrio, and Helicobacter mucosus	Zhu et al. (2022)
		Promotes the production of BAs, SCFAs, and tryptophan metabolites		
		Improves disorders of glucose–lipid metabolism to combat obesity		
Ginseng	Ginsenoside	Increases SCFA levels	Increased: Proteobacteria and Bacteroidetes Decreased: *Verrucomicrobia* and the ratio of *F*/B	Zhuang et al. (2021)
		Intervenes in obesity by regulating the composition of the intestinal flora, improving glycolipid metabolism and inflammatory responses		
	Ginseng pectin	Regulates intestinal flora	Increased: Akkermansia, Bifidobacterium, Bacteroides, and Prevotella	Ren et al. (2023)
		Increases levels of acetic, propionic, and butyric acids and of valine		
		Activation of the AMPK pathway, improving dyslipidemia and obesity		
	White ginseng (WEWG)	Improves gut dysbiosis to fight obesity	Increased: Lactobacillus and Parabacteroides Decreased: *F*/B ratio, *Ruminiclostridium*	Zhou et al. (2020)
	Red ginseng (WERG)	Improves intestinal flora dysbiosis to fight obesity	Increased: Bifidobacterium, Lactobacillus, Akkermansia, and Gastrococcus Decreased: Desulfovibrio and *Escherichia coli*	Guo et al. (2015), Zhou et al. (2020), Peng et al. (2021), Lee et al. (2022), Eun et al. (2023)
		Increases probiotic content for anti-aging effects		
		Improves the structure and composition of the intestinal microbiota to reduce the inflammatory response		
Astragalus	Astragalus polysaccharide (APS)	Improves immunity by improving gut microbiota and increasing body weight and immune organ indices	Increased: Parasutterella, Parabacteroides, Clostridium perfringens XIVb, Butyricicoccus, Dorea, Lactobacillus, Bifidobacterium, Rousselaeria, and Desulfovibrio Decreased: Pseudoflavonifractor, Parapovella, Tyzzerella, and Lachnoclostridium	Li et al. (2023), Wei et al. (2023), Zhao et al. (2023)
		Improvement of gut microbiota, lowering of IL-1β, IL-6, and endotoxin levels, and inhibition of the TLR4/NF-κB pathway to reduce inflammatory responses		
		Modulates gut microbial abundance, increases SCFA production, and promotes anti-inflammatory bacteria		
	Fermented Astragalus	Intervenes in the inflammatory state by regulating the balance of Th1/Th2/Th17/Treg-related cytokines	Alters the structure of the intestinal microbiota and enriches *Akkermansia* and *Aristichthys* species	Li et al. (2022c)
Atractylodes macrocephala	Atractylodes macrocephala volatile oil	Reduces inflammatory stimuli	Increased: Enterorhabdus, Parvibacter, and Akkermansia Decreased: Turicibacter, Parasutterella, and Erysipelatoclostridium	Cheng et al. (2023)
	Atractylodes polysaccharide	Increases the abundance and diversity of gut microbiota	Increased: Relative abundance of potentially beneficial bacteria, such as *Bifidobacterium bifidum* Decreased: Proportion of harmful bacteria, such as *Clostridium strictum* 1 and *Shigella coli*	Kai et al. (2022)
		Reduces proinflammatory cytokine overexpression to achieve anti-inflammatory effects		
		Decreases systemic LPS	Increased: Lactobacillus and Rhodococcus Decreased: *Firmicutes*, *Clostridium difficile*, and *Escherichia coli*	He et al. (2023b)
		Increases levels of tryptophan metabolites		
		Regulates the composition of intestinal flora		
		Regulates disorders of glucose and lipid metabolism		

Further, a computerized search of clinical cases related to the treatment of macular edema in the Chinese full-text journal database and Wanfang database ranked *Poria* in first place, with a recurrence rate of 60 times. Kim et al. (2018) reported improved visual acuity and disappearance of retinal and optic disk hemorrhages in patients with non-proliferative glycoconjugate network after administration of *Poria*-containing Modified-Goshajinkigan (Niucheshenqiwan in Chinese). Lan et al. (2023b) found that *Poria* selectively modulated the abundance of *Odoribacter*, *Muribaculum*, *Oscillibacter*, *E. coli*, and *Turicibacter*, leading to a reduction of the inflammation levels caused by dextran sodium sulfate (DSS) in mice. *Poria* aqueous extract improves metabolic homeostasis and inflammation by restoring intestinal homeostasis, controlling the relationship between GM and host metabolites, modulating hypothalamic neurotransmitters, decreasing proinflammatory cytokines, and inhibiting the expression of TNF-α/NF-κB signaling pathway proteins. Additionally, *Poria* can improve AMD risk factors, such as hyperlipidemia and obesity, by regulating intestinal bacterial communities.

Moreover, water-insoluble *Poria* polysaccharides could improve intestinal mucosal integrity by regulating the intestinal bacterial community and increasing the butyrate-producing biomass of *Clostridium perfringens* (Sun et al., 2020a), thereby promoting glucose-stimulated lipid metabolism, alleviating hyperlipidemia and reducing inflammation and steatosis. Zhu et al. (2022) investigated the effects of *Poria* oligosaccharides on glucose and lipid metabolism disorders in HFD-induced obese mice. Compared to controls, *Poria* oligosaccharides improved insulin resistance and glucose intolerance and reduced insulin and blood glucose levels in HFD-fed mice. Additionally, *Poria* oligosaccharides treatment inhibited the mRNA expression of fatty acid synthesis regulators in epididymal fat and the expression of proinflammatory factors, such as TNF-α, IL-1β, IL-6, and MCP-1. Moreover, *Poria* oligosaccharides partially rectified the imbalance of GM in HFD-fed mice, accompanied by decreased various gut metabolites of significant importance in impairing the intestinal barrier, such as BAs, SCFAs, and tryptophan.

#### 4.2.2 *Panax ginseng* C.A.Mey. [*Araliaceae*; ginseng radix]

*Ginseng*, derived from the desiccated root and rhizome of *Panax ginseng* C. A. Meyer, a member of the Wujiaceae family, possesses several health benefits. It comprises a diverse range of active components, including ginsenosides, polysaccharides, and volatile oils (Su et al., 2023; Zhou et al., 2023), significantly affecting immunity, oxidative stress, inflammation, apoptosis, and coagulation (Zhou et al., 2023). Lee et al. (2015) utilized the ginsenoside targeted transport pathway to improve nutrient exchange in Bruch’s membrane of human donors, delaying its aging and preventing the onset and progression of AMD. Betts et al. (2012) reported that ginsenoside Rb1 from *ginseng* root extract increases the number of cultured adult ARPE-19 cells while decreasing the release of the angiogenic factor VEGF produced by ARPE-19 cells, suggesting that Rb1 plays a role in the prevention of angiogenic ophthalmopathies such as AMD. Cho et al. (2001) demonstrated that ginsenosides inhibited TNF-α production in mouse or human macrophages stimulated by LPS, suggesting that *ginseng* has a preventive effect on AMD.

Additionally, *ginseng* ameliorates AMD risk factors by modulating gut microbes. The ginsenoside Rg5 significantly lowered the *F/B* ratio and markedly attenuated inflammatory responses caused by metabolic endotoxemia. Moreover, Rg5 markedly decreased the abundance of *Firmicutes* and *Verrucomicrobia*. It augmented the abundance of *Proteobacteria* and *Bacteroidetes* in a mouse model of diabetes at the phylum level, ameliorating diabetes-associated dysbiosis and metabolic disorders of the intestinal microbiota. *Ginseng* saponins inhibit obesity and its complications by improving the metabolism of endogenous substances in the gut, reducing inflammation, and altering the composition of the GM. *Ginseng* pectin, a mixed pectin containing the structural domains of rhamnogalacturonan-I and homogalacturonan, improves the intestinal flora by increasing *Bifidobacterium, Akkermansia, Prevotella*, and *Bacteroides,* which increases the levels of propionic, acetic, and butyric acids and valine. These, in turn, participate in cinnamonoside-, 10-hydroxy-8-fen-2-fenuglone glucoside-, leucovorin-, 24-propylcholesterol-3-ol-, and other lipid regulation-related pathways in serum metabolite alterations, and activation of the AMP-activated protein kinase pathway, to ameliorate lipid disorders in obese rats (Zhuang et al., 2021; Ren et al., 2023).

Processed *ginseng* is categorized into white (dried *ginseng*) (WEWG) and red (steamed *ginseng*) (WERG). In HFD-induced obese mice, aqueous extracts of WEWG and WERG demonstrated the ability to alleviate intestinal dysbiosis and exhibited anti-obesity effects, particularly the aqueous extract of WEWG. WEWG markedly decreased the *F/B* ratio and the amount of *Ruminiclostridium* while augmenting the amount of *Parabacteroides* and *Lactobacillus*. WER*G* decreased the amount of *Desulfovibrio* and augmented the amounts of S24-7 and *Gastrococcus* (Zhou et al., 2020). A separate *in vitro* experiment demonstrated that WERG stimulated the growth of the probiotics *Lactobacillus* and *Bifidobacterium bifidum* and curbed the overgrowth of *E. coli*, thereby enhancing the microbiological structure of the intestinal tract and reducing *in vivo* inflammation (Guo et al., 2015). Peng et al. (2021) revealed that WERG treatment caused a more pronounced enhancement of probiotic levels, such as those of *B. bifidum* and *Akkermansia*, than WEWG, indicating the increased anti-aging efficacy of WERG.

Additionally, research on elderly Korean women showed that the consumption of fermented red *ginseng* (RG) modified 20 distinct bacterial species, thereby enhancing overall wellbeing via its impact on defecation, biochemical parameters, and metabolism (Lee et al., 2022). Eun et al. (2023) found that the addition of WERG and its subsequent microbial conversion via the fermentation of RG resulted in alterations in the composition of the GM, exhibiting characteristics of both RG and fermented RG, which were linked to an improved obesity phenotype and glucose balance. These modifications were also linked to the enhanced integrity of the gut barrier, thus safeguarding against inflammation caused by heart failure at both local and systemic levels.

#### 4.2.3 *Astragalus mongholicus* Bunge [*Fabaceae*; astragali radix praeparata cummelle]

*Astragalus* is the dried root of *Astragalus membranaceus*. Its main components are polysaccharides, saponins, flavonoids, amino acids, and other compounds. Additionally, it contains alkaloids, glucuronic acid, iodine, silicon, zinc, and other trace elements. *Astragalus* polysaccharide (APS) (Zheng et al., 2020) and astragaloside (Yang et al., 2022) are the main active components of *Astragalus*. It has antioxidant, anti-inflammatory, anti-aging, anti-apoptosis, immunomodulatory, intestinal mucosa protective, and intestinal flora regulatory effects and modulates signaling pathways (Zheng et al., 2020; Liang et al., 2023). Li et al. (2002) studied a mouse model of retinal photoreceptor degeneration induced by exposure to bright light and the DNA alkylating agent methyl methanesulfonate. They found that astragaloside A reduced the expression of genes involved in necroptosis and inflammatory responses, inhibited microglia activation, and attenuated retinal oxidative stress and inflammation, thereby protecting photoreceptor cells. Similarly, Sun et al. (2020b) discovered that ultrasmall astragaloside-loaded lipid nanocapsule eye drops improved retinal morphology and function in sodium iodate (NaIO3)- induced dry AMD mice and protected retinal function from oxidative stress and apoptosis.

*Astragalus* can exert anti-inflammatory, antioxidant, and immunomodulatory effects by regulating GM abundance. APS altered its composition, enhancing the variety of GM, leading to the reduction in the relative prevalence of *Pseudoflavonifractor* and *Parapovella* and the increase of *Parabacteroides, Parasutterella, Butyricicoccus, Clostridium perfringens* XIVb, and *Dorea*. APS also increased the immune organ indexes and body weights, reduced IL-6, IL-1β, and endotoxin levels, inhibited the TLR4/NF-κB pathway, and improved immune disorders in rats via modulating their GM, particularly certain bacteria implicated in immune and inflammatory responses and production of SCFAs (Zhao et al., 2023). Wei et al. (2023) found that APS regulated GM abundance, enhanced the abundance of anti-inflammatory bacteria, generation of SCFAs, reversed the aberrant expression of NF-κB, NrF2, and their downstream factors in the brain-gut axis, and exerted anti-inflammatory and antioxidant effects on neuronal cells. Li et al. (2023) found that APS significantly activated intestinal TLR4 and MAPK pathways, reversing intestinal flora disorders in immunocompromised mice. APS resulted in an increase in bacterial populations at the genus level, including *Lactobacillus, Bifidobacterium, Rousselaeria*, and *Desulfovibrio*, and a decrease in the abundances of *Tyzzerella* and *Lachnoclostridium*. However, APS did not enhance the immune system of immunocompromised mice with GM depletion. Li et al. (2022c) discovered that supplementing mice with fermented *Astragalus* (FA) controlled abnormal activation of the intestinal immune barrier, resulting in decreased levels of MPO and immunoglobulin E and increased levels of immunoglobulin A. The levels of TNF-α, IL-1β, IL-6, and IL-17 were downregulated in the intestines, and those of TGF-β and IL-10, which are anti-inflammatory, were increased, indicating that FA may affect the inflammatory process by controlling Th1/Th2/Th17/Treg-related cytokines.

Furthermore, fatty acid supplementation modified the composition of the GM and enhanced the presence of *Akkermansia* and *Aristochthys* spp., both of which were associated with the generation of SCFAs. Fermented *Astragalus*-induced microorganisms and their metabolites play a role in preserving the intestinal mucosal barrier’s integrity by affecting intestinal mucosal immunity. Mice supplemented with FA exhibited greater expression of intestinal tight junction protein and mucous-secreting protein ZO-1 and occludin, as well as MUC2 genes, than those supplemented with unfermented *Astragalus*. Repair of the intestinal mucosal barrier by FA has been validated by regulating apoptosis in intestinal epithelial cells Li et al. (2022c).

#### 4.2.4 *Atractylodes macrocephala* Koidz. [*Asteraceae*; atractylodis macrocephalae rhizoma]

*Atractylodes macrocephala*, commonly referred to as “Yuzhu,” “Xizhu,” or “Wuzhu,” functions as the desiccated rhizome of *A. macrocephala* within the Asteraceae family. *Atractylodes macrocephala* contains sesquiterpenes, lactones, polysaccharides, flavonoids, and other chemical components that have antioxidant, anti-apoptosis, and anti-inflammatory effects, improve gastrointestinal function and immunity, and lowers blood lipids (Xie et al., 2023). In a study involving a macular edema formula containing *A. macrocephala*, the total volume of the macular center area in diameter of the patients was significantly reduced, and the visual acuity and visual field were significantly improved (Zhu et al., 2016). In addition, *Atractylodes* may improve AMD risk factors by modulating gut microbes to act as an anti-inflammatory and lipid-lowering agents. *Atractylodes macrocephala* volatile oil reduces harmful bacteria, such as *Parasutterella, Turicibacter*, and *Erysipelatoclostridium*. It increases the number of beneficial bacteria, such as *Parvibacter, Enterorhabdus,* and *Akkermansia*, attenuating the inflammatory response in DSS-induced mouse models (Cheng et al., 2023). Kai et al. (2022) found that *Atractylodes* polysaccharide modulates the intestinal flora by increasing the abundance and diversity of intestinal flora, decreasing the proportion of harmful bacteria, such as *Clostridium strictum* 1 and *Shigella coli*, and increasing the relative abundance of potentially beneficial bacteria, such as *B. bifidum*, thereby alleviating DSS-induced weight loss. It also prevented the overexpression of proinflammatory cytokines TNF-α, IL-1β, and IL-6, reduced neutrophil infiltration, and upregulated the expression of MUC2 and the tight junction protein claudin-1, thus attenuating DSS-induced intestinal mucosal barrier damage in mice. In a rat model administered *A. macrocephala,* He et al. (2023b) observed an abundance of *Lactobacillus* and *Rhodococcus* species in the intestine, as well as an increase in tryptophan metabolites, such as indole, indole-3-propionic acid, and tryptophan. This, in turn, increased the expression of the intestinal AHR. The activation of AHR caused the upregulation of GLP-1 and IL-22 at proteins and mRNA levels, decreased systemic LPS, improved gut barrier function, activated the hepatic STAT3/IL-22R/peroxisomal acyl-coenzyme A oxidase 1 (ACOX1) and pancreatic GLP-1R/p-CREB signaling pathways, and improved lipid metabolism and insulin resistance.

### 4.3 Botanical drug decoction

#### 4.3.1 Shenling Baizhu powder

Shenling Baizhu powder comprises *ginseng*, *Poria*, *A. macrocephala*, *Glycyrrhiza glabra* L. [Fabaceae; radix et rhizoma glycyrrhizae], *Dioscorea polystachya* Turcz [Dioscoreaceae; dioscoreae rhizoma], *Coix lacryma-jobi* var. ma-yuen (Rom.Caill.) Stapf [Poaceae; coicis semen], *Nelumbo nucifera* Gaertn. [Nelumbonaceae; lotus seed], *Lablab purpureus subsp.* Purpureus [Fabaceae; semen lablab album], *Wurfbainia villosa* (Lour.) Skornick. & A.D.Poulsen [Zingiberaceae; amomi fructus], and *Platycodon grandiflorus* (Jacq.) A.DC. [Campanulaceae; platycodonis radix]. Recent research has shown that this powder possesses anti-inflammatory, antiviral, and antioxidative properties, improves immune function, regulates blood lipids, balances intestinal flora, and safeguards the intestinal mucosal barrier. *Ginseng* and Atractylodes Maculatus San protect the blood-retinal barrier, inhibit the expression of inflammatory factors, balance the ecological stability of the intestinal tract by regulating the intestinal bacterial flora, and improve the risk factors for macular edema (Bai et al., 2023). Polysaccharides of Shenling Baizhu powder alleviate inflammation by regulating tryptophan metabolism in *Bacteroides, B. bifidum*, and *Ruminococcus*. The tryptophan metabolite kynurenine activates the expression of polypeptide 1 (CYP1A1) and AHR and promotes the expression of anti-inflammatory IL-10 (Lv et al., 2022). Botanical drugs that strengthen the spleen and benefit qi have demonstrated a significant impact in regulating intestinal flora and enhancing immunity in animal models of spleen deficiency and clinical studies (Lima-Fontes et al., 2022). Shenling Baizhu powder has the potential to enhance the prevalence of the SCFA-producing bacteria *Puccinia* and *Treponema* and decrease the prevalence of the opportunistic pathogens *Vibrio desulfuricans* and *Cholera* spp. It significantly reduces the level of myeloperoxidase, increases the levels of catalase and SOD in the serum of rats, and exerts antimicrobial effects by enhancing the antioxidant capacity and modulating the intestinal microbiota (Gu et al., 2021).

## 5 Conclusion

The human gut is home to hundreds of millions of bacteria and is assumed to be the “second human genome.” Presently, an unhealthy lifestyle and the improper use of antibiotics can result in intestinal flora dysbiosis. The disruption of the GM and alterations in metabolites, including LPS, oxysterols, SCFAs, and BAs, have been linked to various factors impacting AMD, including oxidative stress, apoptosis, aging, inflammation, dyslipidemia, obesity, and modified choroidal hemodynamics.

Exploring the relationship between TCM and GM could be highly beneficial. This investigation could provide insight into how TCM works to prevent and manage AMD, thus broadening the scope of TCM theory. In recent years, there have been fewer reports of TCM treating AMD by modulating GM. This review focused on the correlation between GM and its metabolites and AMD-related risk factors, drawing upon previous research. Furthermore, we demonstrated the possible ways TCM modulates the GM to improve AMD based on classification. While organizing the data, we found that the diversity of intestinal flora may be associated with host species, dietary preferences, and lifestyle habits. Consequently, we hypothesized that different parts of the intestinal tract harbor different types and amounts of dominant GM within the same individual. However, the relationship between various gut bacteria and the precise mechanisms by which the GM influences AMD remain unclear. Further studies are required to identify the precise pharmacodynamics, target pathway, and mechanism of action of TCM. Consequently, future research should concentrate on the biotransformation of TCM active components via the GM and whether these biotransformed metabolites positively or negatively influence TCM’s efficacy in treating AMD. Further animal trials and clinical randomized, controlled, multicenter, large-sample studies should be conducted to confirm the effectiveness and safety of TCM in treating AMD via GM regulation and to identify novel therapeutic objectives for AMD.

## Scope statement

This review focuses on the mechanism of action of Chinese medicine in treating AMD, as well as previous related research. Literature suggests that gut flora-related mechanisms may be a plausible explanation. All these are related to the fields of ethnopharmacology, intestinal flora, molecular mechanisms, and neurodegenerative diseases, which are the focus of your journal, and we hope to communicate with and learn from the general readers of your journal through your journal.
